# Supplementary material for: ChatGPT-generated help produces learning gains equivalent to human tutor-authored help on mathematics skills
Source: PLoS One. 2024 May 24;19(5):e0304013. doi: 10.1371/journal.pone.0304013 (PMC11125466; doi:10.1371/journal.pone.0304013)
Supplement: Attachment — Submitted filename: ChatGPT Math Hint PLOS response (2).pdf [file pone.0304013.s001.pdf]

Dear Editor,

We greatly appreciate the feedback of reviewers and have detailed below how we have made revisions to the manuscript in response to each raised point.

Reviewer #1:

1. Consider adding a sentence or two at the start of the abstract to briefly introduce the concept of "authoring."

   We have added that authoring requires “many iterations of content creation, refining, and proofreading” to highlight stages of the authoring process.

2. For the "3x4 study design", it would be beneficial to clarify the conditions or factors. Are the three conditions ChatGPT, human tutor, and no-help control, and the four conditions refer to distinct mathematics subjects? If so, kindly specify this in the paper.

   We have addressed this by adding the sentence, “Participants are randomly assigned to one of three hint conditions (control, human tutor, or ChatGPT) paired with one of four randomly assigned subject areas (elementary algebra, intermediate algebra, college algebra, or statistics).”

3. The motivation of this research is unclear. What is the motivation to answer the proposed two research questions? The first and second paragraphs in Introduction basically described LLM and then the third paragraph described the experimental process. Why is worthwhile to answer two research questions? Additionally, what is the connection between RQ1 and RQ2?

   We have modified the paragraph after the introduction of the Research Questions (RQs) to explicitly address why it is worthwhile to answer each research question. We hope that this modification also makes clear the motivation of the work:

   “If ChatGPT, or other LLM-generated hints are found to have sufficiently low error (RQ1) and sufficiently high learning efficacy (RQ2), it would alleviate the most time-constrained and cost-intensive component of tutoring system development and open the door to previously unrealized scaling of these types of interventions in a multitude of domains and learning contexts.”

4. The term "tutor authoring tools" lacks a clear definition or description in the introduction. Given that Plos ONE is a multidisciplinary journal, it's essential to define such terms for broader readers.

We have further clarified the term “tutor authoring tools,” by giving an example and citation to “CMU’s Cognitive Tutor Authoring Tools (CTAT)” and clarifying that we are referring to “tutoring *system*” tools.

5. Please describe on the "hallucination mitigation technique" in the section of introduction.

   We have added the following paragraph to the introduction before the Research Questions to define the mitigation technique:

   “Large Language Models have been known to "hallucinate," sometimes producing results that are factually incorrect [13]. Such errors contained in ChatGPT-generated hints could have detrimental effects on learning. We therefore evaluate ChatGPT-generated hints for the presence of hallucinations and experiment with an error mitigation technique called self-consistency [14], which calls for prompting the model many times and keeping the modal response.”

6. I feel the section of Related Work is more like introducing the Background of Automatic hint generation and ChatGPT development and early applications. Would be good if authors could discuss the recent progress of ChatGPT in education from the community of EDM, AIED, LAK, L@S and etc, and the research gap related to the submitted work.

   We agree that the Related Work is in need of a ChatGPT in Education section given recent developments in that area. We’ve added the following new related work section, which includes recommended citations given by Reviewers 1 and 3, as well as other relevant citations:

   “Initial reactions to ChatGPT in education centered around the potential increase in cheating threatening existing assessments, particularly in higher education [4, 41]. This was followed by more optimistic speculations of its use in bolstering educational outcomes [42–44], providing personalized learning experiences [45, 46], and supporting instructors [47, 48]. Since then, empirical research has evaluated the ability of ChatGPT to provide meaningful feedback to wrong answers in a decimal point learning game [49], fielding students’ questions on programming assignments [50], evaluating crowdsourced multiple-choice questions [51], and improving the relevance of ChatGPT’s answers to educational questions by including contextual textbook information [52]. In all of these examples, errors or hallucinations were observed, underscoring the importance of taking a critical approach to evaluating LLM outputs in an educational setting [53].”

   None of the related work in the field conducts a randomized controlled study to measure a ChatGPT implementation’s effect on learning outcomes and none compare to a human or human-authored control. Our work addresses this research gap.

7. "Nascent works have conducted offline evaluation of GPT-3 [16]", why authors discussed the evaluation of GPT-3. There are bunch of research evaluated the capability of ChatGPT (GPT-3.5) [1,2,3] in educational field. Also, the main focus of this paper is about ChatGPT.

   We have changed the word "nascent" to "past." Thus, the new sentence is: "Past works have conducted offline evaluation of GPT-3 [18]." Additionally, all the papers suggested by the Reviewer have now been added to a new "ChatGPT in Education" related work section.

8. The experimental design and data analysis appear thorough. However, can authors justify the choice of their prompting strategies? and also please put the details of prompting strategies in the paper, which would largely help the researchers from the community to repeat this study.

   We discuss this in our "Prompt engineering" subsubsection within the method section. In this subsubsection, we have added details to our prompt engineering process, stating, "in exploratory use of ChatGPT, we found no special prompting was required to elicit the desired worked solution from ChatGPT. ChatGPT seemed to be trained/fine-tuned to be naturally verbose." We have also added an example of a prompt to this section corresponding to the prompt used to generate the hint for Figure 2.

9. How did authors determine the quality (low and high) of hints? By human expert? Any evaluation rubric? if the quality of hints are determined by the correctness (incorrect and correct), why not just say incorrect and correct?

   In our "Quality checks" subsubsection in the method section, we mention the use of "a three point check to ensure that 1) the correct answer was given in the worked solution 2) the work shown was correct and 3) that no inappropriate language was used. A hint was considered fully correct if it met these three criteria." Thus, a hint was deemed as high quality if it met these three criteria. If it failed to meet any of these checks, it was deemed as low quality, not just the correctness check.

10. What is the implication/practicality of the findings from RQ1?

    Based on the 26.66% error rate, we have added the following to the manuscript related to implications:

    "This all suggests that ChatGPT and other LLMs should not be used to give feedback to students in the same way a teacher or teaching assistant would, unless it was in a domain verified to have near zero error. If error mitigation cannot reduce the error to near zero, system designers should consider framing ChatGPT-produced feedback as coming from an "imperfect robot" or peer-like source of information so that students may consider its responses critically."

11. There are several "P<<0.001" throughout the paper. Do the authors mean "p<0.001"?

    The double less than sign is used to express that a value is much less than another value. There is a similar looking single character symbol that explicitly means “much less than” which we are now using instead of the double less than.

12. On page 9/14, the correct phrasing should be "necessary conditions have been met."

    This change has been made in the manuscript.

13. With the release of GPT-4 in early March 2023, the experiment date of the submitted study is in February, would GPT-4 provide different or enhanced results?

    In the reviewed paper, we note that Open AI’s own paper on ChatGPT 4 applied to college-level science and math topics exhibits a similar error to what we observed using 3.5. We have, however, now added that “ChatGPT 4 may be expected to exhibit more significant improvements from 3.5 on high school-level math, with a reported 19 raw percentile reduction in error on SAT Math [58].”

Reviewer #2:

1. Line 2: Remove the term "first" from "first efficacy evaluation" since it is difficult to ascertain if this study is truly the first in the rapidly evolving research field.

    We have removed the term "first" from "first efficacy evaluation."

2. The term "human tutor" is unclear. In Line 145 to 154, the authors discuss the "Human Tutor Hint Generation" – OATutor system, but it is unclear if "human tutor" refers to this system or an actual person. Clarification is needed, and if the reference is to a real person, their contribution and the quality of hints should be outlined.

    “human tutor” refers to the hints within the OATutor system that were authored by humans. This acts as a contrast from ChatGPT-generated hints which were AI based. We’ve clarified this by stating, “human tutor-authored help”

3. In the Experimental design section (Line 223 to 240), details about student activities in each research group should be included to provide a clear understanding of what happened in each group.

    We highlight the activities in the experimental design. The only differences between the phases were the five question hint condition sequences. The sequences were either the human tutor-authored hints (with an example shown in Figure 1), ChatGPT-generated

hints (with an example shown in Figure 2), or no feedback. Since we had not elaborated on the no feedback condition, we added the following sentences to clarify this: "The control condition did not differ from the other conditions, except for the absence of hints. Participants in the control still received correctness feedback during the "acquisition phase" and could ask for the "bottom out hint" which gave them an answer they could copy and paste into the submission box to move on. Participants in the other conditions had access to the full worked solution in addition to this bottom out hint."

4. Specify the version of GPT used in the experimental design to enhance clarity and completeness.

   We have explicitly added "the 'Dec 15 Version' of ChatGPT 3.5 was prompted to generate hints in the experiment condition" at the end of the experimental design section

5. Address the missing analysis regarding RQ1 (Line 259 to 269). How was the quality of ChatGPT hints rated, and what was the interrater reliability? Explain the absence of a similar evaluation for Human tutor hints.

   In our "Quality checks" subsubsection in the method section, we mention the use of "a three point check to ensure that 1) the correct answer was given in the worked solution 2) the work shown was correct and 3) that no inappropriate language was used. A hint was considered fully correct if it met these three criteria." Since this check was relatively objective (i.e., if steps evaluate to a correct answer and if inappropriate language was present), we did not employ multiple raters as would be expected if the content were being rated for quality on a more subjective scale.

6. Clarify the activities or interventions implemented in the control group and explain the unusual gains and losses observed in terms of elementary math, college math, and statistics for the control group students as presented in Table 2.

   We address both the Reviewer's points with the addition of the following sentences:

   "The control condition did not differ from the other conditions, except for the absence of hints. Participants in the control still received correctness feedback during the "acquisition phase" and could ask for the "bottom out hint" which gave them an answer they could copy and paste into the submission box to move on. Participants in the other conditions had access to the full worked solution in addition to this bottom out hint. The three negative learning gains were associated with control conditions but were not statistically significant and therefore likely not indicative of actual knowledge loss."

7. Suggest including p-values in the table for informative purposes to enhance the clarity of the presented results.

We have included p-values in the table for informative purposes to enhance the clarity of the presented results.

Reviewer #3:

1. I believe the authors did a reasonable job of highlighting this importance, but it would be useful if the authors could better articulate why their specific study is necessary. Specifically, how does this work relate to other recent similar studies by Arto Hellas and others.

   We have modified the paragraph after the introduction of the Research Questions (RQs) to explicitly address why it is worthwhile to answer each research question:

   "If ChatGPT, or other LLM-generated hints are found to have sufficiently low error (RQ1) and sufficiently high learning efficacy (RQ2), it would alleviate the most time-constrained and cost-intensive component of tutoring system development and open the door to previously unrealized scaling of these types of interventions in a multitude of domains and learning contexts."

   We have added a third subsection dedicated to ChatGPT applications in education. In this third subsection, we have cited the mentioned paper. That work and ours evaluated the "quality" of ChatGPT feedback. Our study distinguishes itself by doing so in mathematics and by measuring the effect of a ChatGPT intervention on learning outcomes using a randomized controlled experimental design and comparing it to a human or human-authored control.

2. Relatedly, the authors would benefit from restructuring their related work. The authors have an entire section dedicated to LLMs but omit a lot of work related to LLMs being applied in educational settings. The authors do include some of most directly relevant related work about generating explanations, but the paper would be strengthened by including addition work such as…

   We have added a third subsection dedicated to ChatGPT applications in education. In this third subsection, we have cited and contextualized the reviewers' suggested related works:

   "Initial reactions to ChatGPT in education centered around the potential increase in cheating threatening existing assessments, particularly in higher education [4, 41]. This was followed by more optimistic speculations of its use in bolstering educational outcomes [42–44], providing personalized learning experiences [45, 46], and supporting instructors [47, 48]. Since then, empirical research has evaluated the ability of ChatGPT to provide meaningful feedback to wrong answers in a decimal point learning game [49], fielding students' questions on programming assignments [50], evaluating crowdsourced multiple-choice questions [51], and improving the relevance of ChatGPT's answers to

educational questions by including contextual textbook information [52]. In all of these examples, errors or hallucinations were observed, underscoring the importance of taking a critical approach to evaluating LLM outputs in an educational setting [53]."

3. Prompting: It was unclear what prompt was used in this study. The authors write extensively about how LLMs work in the "Model" section, but it would be more useful to focus on how the models were prompted. Given the significant impact that prompting has on model performance, it would be useful to see how performance varied across multiple prompts or at least there should be a solid justification for why the prompt was chosen.

   We discuss this in our "Prompt engineering" subsubsection within the method section. In this subsubsection, we have added additional information about the prompt:

   "In exploratory use of ChatGPT, we found no special prompting was required to elicit the desired worked solution from ChatGPT. ChatGPT seemed to be trained/fine-tuned to be naturally verbose." We have also added an example of a prompt in this section.

4. Sampling: Excluding 30.4% of participants is a large amount. The authors claim that these rates are consistent with prior work, but that prior work (e.g.: Simon and Walker) conducted a multi-day study where attrition is expected to be higher. Given this was a short 20 minute study done in one session, this connection to prior work is a false equivalence. Authors should provide better justification or revise recruitment.

   To avoid the false equivalence the Reviewer speaks of, we have changed our wording from characterizing the 30% as "anticipated" to instead characterizing it as high but not unprecedented for published work using MTurk.

   Furthermore, we have added the 30% as a limitation to the limitations portion of our manuscript and added better justification for why this attrition is not a threat to the main learning gain comparison results of the paper (RQ2):

   "We observed a high attrition rate of 30% in our study. However, this attrition was fairly even across conditions (i.e., 36-43 excluded participants) with the experiment condition (i.e., ChatGPT) having the fewest excluded participants. Because of the relative evenness of attrition across conditions, it is unlikely to have posed a significant threat to the validity of between condition comparisons."

5. Sampling Bias: Another concern around sampling is the variability in pre-test performance. The authors had a good idea to exclude participants that didn't have a high school background to partially account for differences, but given that some participants might not have taken algebra (or prereqs) in decades introduces some confounds. This is one of many confounds related to working with crowd workers in this learning context. The authors need to do a better job of justifying why crowd workers were used instead of students in an algebra class.

We have added the use of crowdworkers as a limitation but include a justification for their use with the following addition:
"Our study utilized crowdsourced learners as opposed to in-situ secondary and post-secondary students. This choice was made in part due to the challenge in gaining access to classrooms, the heightened risk factor considered by both a research ethics board and teachers when considering an experiment using such nascent technology, and the need for a learner participant pool allowing for a speed of experimentation that can attempt to keep up with the speed of AI technology iteration. While we conclude that our crowdsourced participants exhibited authentic learning, effects may differ in classroom settings with students learning the material for the first time as part of their curriculum."

6. Questions: Providing more details about pre and post-test questions, as well as the learning task, would enhance the method section. How did the authors ensure that participants engaged with the questions and hints?

   To ensure that participants engaged with the questions and hints, we checked that they had attempted all questions by consulting our log data. Crowd workers are also generally motivated to diligently complete a task to not be disqualified from payment afterwards. Additionally, the percent correct rate and learning gains suggest that even though we didn't necessarily apply additional measures to ensure engagement, learning gains are evidence that there was engagement with the questions and hints. We also only included participants who had answered all the questions in our study which ensured that our analysis only included participants that were sufficiently engaged.

7. Hints: Describing whether incorrect ChatGPT hints were shown, along with details on hint differences (e.g.: length, content, structure, etc) between conditions, is necessary for interpreting learning gains.

   Incorrect ChatGPT hints were not shown. In our "Quality checks" subsubsection in the method section, we mention the item exclusion criteria and clarify by adding that this is a "screening" process:

   "a three point check to ensure that 1) the correct answer was given in the worked solution 2) the work shown was correct and 3) that no inappropriate language was used. A hint was considered fully correct if it met these three criteria. If a hint failed to meet even one of these criteria, the question it was associated with was disqualified, resulting in a decrease in the pool of questions that could be utilized for the experiment."

   We conducted a length analysis on the ChatGPT-generated hints vs. human tutor-authored hints as suggested by the reviewer:

   ChatGPT-generated hints - Min: 142, Median: 355, Max: 699

Human tutor-authored hints - Min: 79, Median: 277, Max: 667

These descriptives are now added to the end of the RQ1 results section

8. Hypothesis Testing: A more robust statistical model considering multiple comparisons and covariates related to hints and participants would strengthen the analysis. Time-on-task should not be evaluated with a separate model but should be included as a variable. The model should ideally also include math subject rather than computing means across conditions directly.

   We agree with the Reviewer that such a statistical model would strengthen the analysis. However, we chose to keep the existing analytical methodology for two reasons, 1) We wanted to be able to directly compare to the results of the previous small scale pilot published on the arXiv pre-print server, as this current study remedies all of the shortcomings identified in that study and it would add conflation to comparison if the current study differed in analytical methodology. 2) As our current methodology has not been claimed to be unsound, even if improvements are available, we did not want to deviate from the pre-defined statistical methodology laid out in the "Analysis" methods section. Doing so may be construed as a form of applying multiple tests until a significant result is found, or "p-hacking"

   Additionally, a benefit of our publicly releasing the dataset is that it allows for the field to conduct alternative analyses of the data.

9. Learning Gains: The disparity in pretest scores among conditions should be addressed, considering the potential influence of the ceiling effect. Given the 6% difference in learning gain, could that be partially explained by ChatGPT participants having performed 10% worse on average in the pre-test?

   Participants in all conditions were even at pre-test within each subject as per a Kruskal-Wallis test ($p = 0.451$ for Elementary Algebra, $p = 0.785$ for Intermediate Algebra, $p = 0.265$ for College Algebra, $p = 0.382$ for Statistics). This is mentioned in the results section of our manuscript.

10. While not influencing my review, a qualitative analysis of hints could provide additional context. What other factors besides correctness (e.g.: length, content, structure, etc) , could differentiate between hint types and affected learning gains?

    We conducted a length analysis on the ChatGPT-generated hints vs. human tutor-authored hints as suggested by the reviewer:

    ChatGPT-generated hints - Min: 142, Median: 355, Max: 699
    Human tutor-authored hints - Min: 79, Median: 277, Max: 667

    These descriptives are now added to the end of the RQ1 results section
