# Supplementary material for: ChatGPT-generated help produces learning gains equivalent to human tutor-authored help on mathematics skills
Source: PLoS One. 2024 May 24;19(5):e0304013. doi: 10.1371/journal.pone.0304013 (PMC11125466; doi:10.1371/journal.pone.0304013)
Supplement: Attachment — Submitted filename: PLOS One Decision Annotations (Round 3).pdf [file pone.0304013.s002.pdf]

Dear Editor,

We greatly appreciate your feedback and that of reviewers and have detailed below how we have made revisions to the manuscript in response to each raised point.

Editor:

1. Inter-rater reliability (IRR) must be performed; ease of rating does not equate to consistency, and we must avoid results being influenced by the individual who conducted the rating.

   We have collected additional data in order to perform IRR and added details of this analysis, including the following: “We evaluated the inter-rater reliability of six UC Berkeley undergraduate raters across all four subjects: Elementary Algebra, Intermediate Algebra, College Algebra, and Statistics. To quantify the agreement among our six raters, we employed Fleiss' Kappa. For all subjects, Fleiss’ Kappa were 0.929 for Elementary Algebra, 0.864 for Intermediate Algebra, 0.857 for College Algebra, 0.916 for Statistics, all showing almost perfect agreement (Landis and Koch, 1977).” Table 1 has also been updated to reflect data gathered from our raters instead.

2. The experiment involved random assignment to different subjects and conditions, implying main effects and interactions that were not analyzed.

   We have addressed this by using a Two-Way ANOVA on a ranked transformation of learning gains to identify the main effects of the condition, as well as to explore any interactions between the condition and the subject variable. We have added the following to our manuscript: “Kruskal-Wallis will also be used to assess the statistical significance from pre- to post-test scores among each subject-condition pairing. For an overall analysis between the human tutor-authored hints, ChatGPT hints, and no hints conditions, a Two-Way ANOVA on ranked data will be conducted to identify the main effects of the condition on learning gains, as well as to explore any interactions with the subject variable. Our analysis will utilize an Ordinary Least Squares (OLS) regression with the model being formulated as follows:

   $$\text{Ranked Gain} = \beta_0 + \beta_1 \times \text{Condition} + \beta_2 \times \text{Subject} + \beta_3 \times (\text{Condition} \times \text{Subject}) + \varepsilon$$

   where: β_0 is the intercept, β_1 captures the effect of different conditions on learning gain, β_2 represents the influence of the subject matter, β_3 estimates the interaction effect, and ε is the error term. If statistical significance is evident, a post-hoc analysis using Dunn's test will be performed for a detailed breakdown of pairwise comparisons.”

   We have also added the following: “Investigating how the conditions compared to one another, we found significant main effects of the condition ($F(2, 262) = 5.037$, $p = 0.0071$) and the subject ($F(3, 262) = 6.737$, $p = 0.0002$), indicating that the type of hints

provided had statistically significant impacts on the learning gains and that there were differing amounts of learning by subject. There was, however, no statistically significant interaction between condition and subject (F(6, 262) = 0.901, p = 0.495). To analyze which conditions were statistically significantly separable from one another, a post-hoc analysis using Dunn's test was performed. From this test, we found that compared to the 1.85\% gain of the control condition (i.e., "no hints"), learners in the ChatGPT condition exhibited statistically significantly greater learning gains (p = 0.011). Human tutor-authored hints were not statistically significantly different from the control (p = 0.087). When comparing the magnitude of learning gain from the human tutor hints and ChatGPT hints, it can be observed that ChatGPT hints produced 46.30\% higher learning gains, overall, as compared to human-authored hints. As seen in Fig \ref{fig:learninggains}, learning gains for all subjects were higher in the ChatGPT condition. However, the ChatGPT and human-authored hint learning gains were not statistically significantly separable (p = 0.416). When conducting pairwise comparisons between subjects, only Elementary Algebra showed statistically significant differences, with higher learning gains exhibited than the three other subjects. Participants in all conditions were even at pre-test within each subject as per a Kruskal-Wallis test (p = 0.451 for Elementary Algebra, p = 0.785 for Intermediate Algebra, p = 0.265 for College Algebra, p = 0.382 for Statistics)."

3. Relatedly, a complete characterization of the power achieved and the impact of the high attrition rate on your conclusions is necessary.

   We have included a characterization of the power achieved by adding the following description: "We conducted a power analysis for each of the pairwise comparisons among the human-tutor hints, ChatGPT hints, and no hints conditions. The power analyses were based on an assumed effect size of Cohen's d = 0.5, a significance level of 0.05, and the sample sizes (90 for Control, 86 for human-tutor hints, and 98 for ChatGPT hints). We found the following power levels: 0.910 for no hints and human-tutor, 0.926 for no hints and ChatGPT, and 0.920 for human-tutor and ChatGPT, indicating that our study was sufficiently powered."

   We have specified the impact of the high attrition rate on our conclusion through the following description: "We observed a high attrition rate of 30% in our study. However, this attrition was fairly even across conditions (i.e., 36-43 excluded participants per condition). Because of the relative evenness of attrition across conditions, it is unlikely to have posed a significant threat to the validity of between condition comparisons."

4. On a minor note, I do not understand what the p-values in Table 2 refer to. Since there are 3 levels in the variable condition, what pair is that p-value referring to and what measure are you comparing?

   The p-values are from a comparison of within subject-condition pre- and post-test scores. We have clarified this in the manuscript by adding the following: "We also

present the p-values obtained from our statistical analysis, comparing pre- and post-test scores across the different subject-condition pairings.” This analysis answers the first part of RQ1 that does not involve cross-condition comparison, “RQ1: Do ChatGPT hints produce learning gains”

Reviewer 3:

1. Regarding the contributions, one persisting issue pertains to the use of the term "human-tutors”. The authors added a short description in the paper clarifying what is intended by the term. However, as presented in the title and elsewhere throughout the paper, this term gives a misleading impression that students interacted with human tutors.

   We have modified the title to replace "human-tutors” with “human tutor-authored help” so as to avoid any misinterpretation that one-on-one, live tutoring was involved. Our modified title is: “ChatGPT-generated help produces learning gains equivalent to human tutor-authored help on mathematics skills.” - Throughout the manuscript we also now refer to human tutor-authored help.

2. Similarly, referring to ChatGPT as "help" in the title gives the impression students could interact with ChatGPT directly and ask questions. Instead, hints were generated with ChatGPT and those hints were used. It is important to note that only correct hints were used.

   We believe that our use of “generated help” sufficiently conveys content being authored by ChatGPT as opposed to an interactive intervention. If readers infer that this could be achieved without human checking of the correctness, this is largely true, as we find that self-consistency ameliorates the errors in three of the four subject areas.

3. Given that the authors acknowledge the limitation posed by studying crowd workers rather than students, the inclusion of "a crowdsourcing study" in the title is suggested to provide transparency about the sample used.

   Recent work (Wang et al., 2024) shows generalizability between educational study results using crowdsourced learners vs in-situ learners. We are explicit about the crowdsourcing methodology in the paper and, due to this generalizability between the sources of the sample, we do not believe a distinction is warranted in the title.

   Recent research, which we have cited in our manuscript, demonstrates the generalizability of results between crowdsourced participants and in-classroom students for an interactive problem-solving task (Wang et al., 2024). They found that crowdsourced learners performed on par with participants from the physics class in terms of obtaining the correct solutions. From this, we believe the insights gained from our study have broader implications beyond crowdsourced learners and can be

generalized. Furthermore, our manuscript clearly outlines the parameters of our recruitment process to ensure that readers are fully aware of the context and can appropriately gauge the applicability of our findings to their own settings.

4. The authors claim it was easy to rate, but IRR is used to ensure others agree with you that it is easy to rate.

   We have collected additional data in order to perform IRR and added details of this analysis, including the following: “We evaluated the inter-rater reliability of six UC Berkeley undergraduate raters across all four subjects: Elementary Algebra, Intermediate Algebra, College Algebra, and Statistics. To quantify the agreement among our six raters, we employed Fleiss' Kappa. For all subjects, Fleiss’ Kappa were 0.929 for Elementary Algebra, 0.864 for Intermediate Algebra, 0.857 for College Algebra, 0.916 for Statistics, all showing almost perfect agreement (Landis and Koch, 1977).” Table 1 has also been updated to reflect data gathered from our raters instead.

5. Methodological decisions are interwoven into the results section, such as the addition of "The control condition did not differ from the other conditions, except for the absence of hints." This integration hinders clarity, forcing readers to search for information and infer details.

   We have moved the following sentences into the experimental design section: “The control condition did not differ from the other conditions, except for the absence of hints. Participants in the control still received correctness feedback during the "acquisition phase" and could ask for the “bottom out hint” which gave them an answer they could copy and paste into the submission box to move on. Participants in the other conditions had access to the full worked solution in addition to this bottom out hint.”

6. There is ambiguity in the discussion of attrition, where the authors mention a high rate but later state ChatGPT had the fewest excluded participants. Clarification is needed on whether participants dropped out (attrition) or were excluded.

   We have removed the sentence about ChatGPT having the fewest excluded participants because attrition was fairly even across conditions (i.e., 36-43 excluded participants per condition). Because of the relative evenness of attrition across conditions, it is unlikely to have posed a significant threat to the validity of between condition comparisons.”

   In our analysis, we only included participants who completed the entire study. Thus, if a participant dropped out, they were excluded from the study; our attrition comes from excluded participants who did not complete the study.

7. Additionally, there are still typos and errors, including missing information in the caption for Figure 4 and a citation on page 416.

These have been fixed. All typos and errors have been corrected.
